# Explore mediated co-varying dynamics in microbial community using integrated local similarity and liquid association analysis

**DOI:** 10.1186/s12864-019-5469-8

**Published:** 2019-04-04

**Authors:** Dongmei Ai, Xiaoxin Li, Hongfei Pan, Jiamin Chen, Jacob A. Cram, Li C. Xia

**Affiliations:** 1 School of Mathematics and Physics, University of Science and Technology Beijing, Xueyuan Road, Haidian District, Beijing, 100001 China; 2 Department of Medicine, Stanford University School of Medicine, 269 Campus Dr., Stanford, CA 94305 USA; 3 Center for Environmental Science, University of Maryland, Cambridge, MA 21613 USA

**Keywords:** Liquid association, Local similarity analysis, Microbial ecology, Time series data, Three-way association

## Abstract

**Background:**

Discovering the key microbial species and environmental factors of microbial community and characterizing their relationships with other members are critical to ecosystem studies. The microbial co-occurrence patterns across a variety of environmental settings have been extensively characterized. However, previous studies were limited by their restriction toward pairwise relationships, while there was ample evidence of third-party mediated co-occurrence in microbial communities.

**Methods:**

We implemented and applied the triplet-based liquid association analysis in combination with the local similarity analysis procedure to microbial ecology data. We developed an intuitive scheme to visualize those complex triplet associations along with pairwise correlations. Using a time series from the marine microbial ecosystem as example, we identified pairs of operational taxonomic units (OTUs) where the strength of their associations appeared to relate to the values of a third “mediator” variable. These “mediator” variables appear to modulate the associations between pairs of bacteria.

**Results:**

Using this analysis, we were able to assess the OTUs’ ability to regulate its functional partners in the community, typically not manifested in the pairwise correlation patterns. For example, we identified *Flavobacteria* as a multifaceted player in the marine microbial ecosystem, and its clades were involved in mediating other OTU pairs. By contrast, *SAR11* clades were not active mediators of the community, despite being abundant and highly correlated with other OTUs. Our results suggested that *Flavobacteria* are more likely to respond to situations where particles and unusual sources of dissolved organic material are prevalent, such as after a plankton bloom. On the other hand, *SAR11*s are oligotrophic chemoheterotrophs with inflexible metabolisms, and their relationships with other organisms may be less governed by environmental or biological factors.

**Conclusions:**

By integrating liquid association with local similarity analysis to explore the mediated co-varying dynamics, we presented a novel perspective and a useful toolkit to analyze and interpret time series data from microbial community. Our augmented association network analysis is thus more representative of the true underlying dynamic structure of the microbial community. The analytic software in this study was implemented as new functionalities of the ELSA (Extended local similarity analysis) tool, which is available for free download (http://bitbucket.org/charade/elsa).

## Background

Marine ecosystem dynamics are likely controlled by environmental conditions and by the interactions between individual microbial taxa in the environment [1, 2]. Therefore, the study of the interplay among microbial species and the factors that control them are critical to understand and predict ecosystems’ functions and their response to environmental changes. But how to apply theoretical approaches to predict species interactions in microbial communities from proximal data such as taxon abundances is still a key challenge in microbial ecology [3, 4]. Studies of microbial co-occurrence patterns [5–7] have led to novel insights into the ecology and habitats of different bacterial species. For instance, correlation network analyses have shown that related organisms, such as different ecotypes of *SAR11* respond to different environmental conditions and co-occur with different bacterial, archaeal and protistan species [7, 8]. Such analyses have also elucidated the differences in the interactions between grazers and viruses with bacteria [9, 10], and have suggested that changes in surface environments can have an effect on bacteria deep in the ocean [11, 12].

One limitation of previous correlation-based methods, such as *CoNet, MENAP*, *SparCC*, and *CCLasso* [13–16] is that they examine only pairwise correlations between organisms. It is however observed that some relationships between organisms are mediated by third variables [17–19]. For example, in marine ecosystems, mixotrophic eukaryotes feed on bacteria under some conditions (such as low sunlight or high prey density) but are photosynthetic under others. The trophic mode of these mixotrophs, is thus influenced by environmental conditions; in turn the way the mixotrophs interact with their environment is influenced by their trophic mode. Thus, a three variable mediation relationship could exist where sunlight levels (through trophic mode) affect the relationship between the mixotroph and its prey. Many bacteria are mixotrophic as well [20–22], and may similarly have interactions with their surroundings that are mediated by the variables that determine the trophic mode.

Other associations that likely include more than two variables would include symbioses that are favorable under only certain conditions. A well-known example is the symbioses between nitrogen fixing bacteria and their eukaryotic hosts [23–25], which are likely only prevalent in nitrogen-limited environments. Syntrophic relationships likewise would only exist under conditions favorable to that relationship, as would predation, competition and any other interaction that could occur between two organisms but might be mediated by third variables.

To examine how co-occurrence might be mediated by biological or environmental variables we employed liquid association (LA) analysis [26, 27] in succession to co-occurrence network analysis by the local similarity analysis (LSA) [6, 28, 29]. The rationale here is that LSA acts as a filtering mechanism to limit our three way analysis to only those variables that were at least sometimes associated with each other. Importantly, local associations, measured by ELSA [28–30], can include patterns that are present and robust over some parts of a time-series data set while weak or absent in other parts of that time-series. A natural question is whether the time-series are associated with a third variable. This question can then be measured by LA.

Our newly implemented Extended Liquid Association (ELA) analysis pipeline integrates both co-occurrence and mediation analysis to identify both pairwise and third party mediated associations. The ELA software is available as subroutines of the ELSA package (http://bitbucket.org/charade/elsa/). The tool will enable researchers to address the co-occurrence phenomenon with additional perspective of changing pairwise species-species or species-environment interaction with regard to a third species or environmental factor, or a varying ecosystem indicator. In this study, we identified multi-party interactions in interspecies and species-environment interaction using the *San Pedro Ocean Time-series* (*SPOT*) microbial community data by *Cram* et al. [11], which profiles the marine ecosystem near Los Angeles coast [7] using *Automated Ribosomal Intergenic Spacer Analysis* (*ARISA*) [31]. We also discussed the limitations of current analysis and possible future developments of ELA.

## Methods

### Analysis scheme

**Fig. 1:**
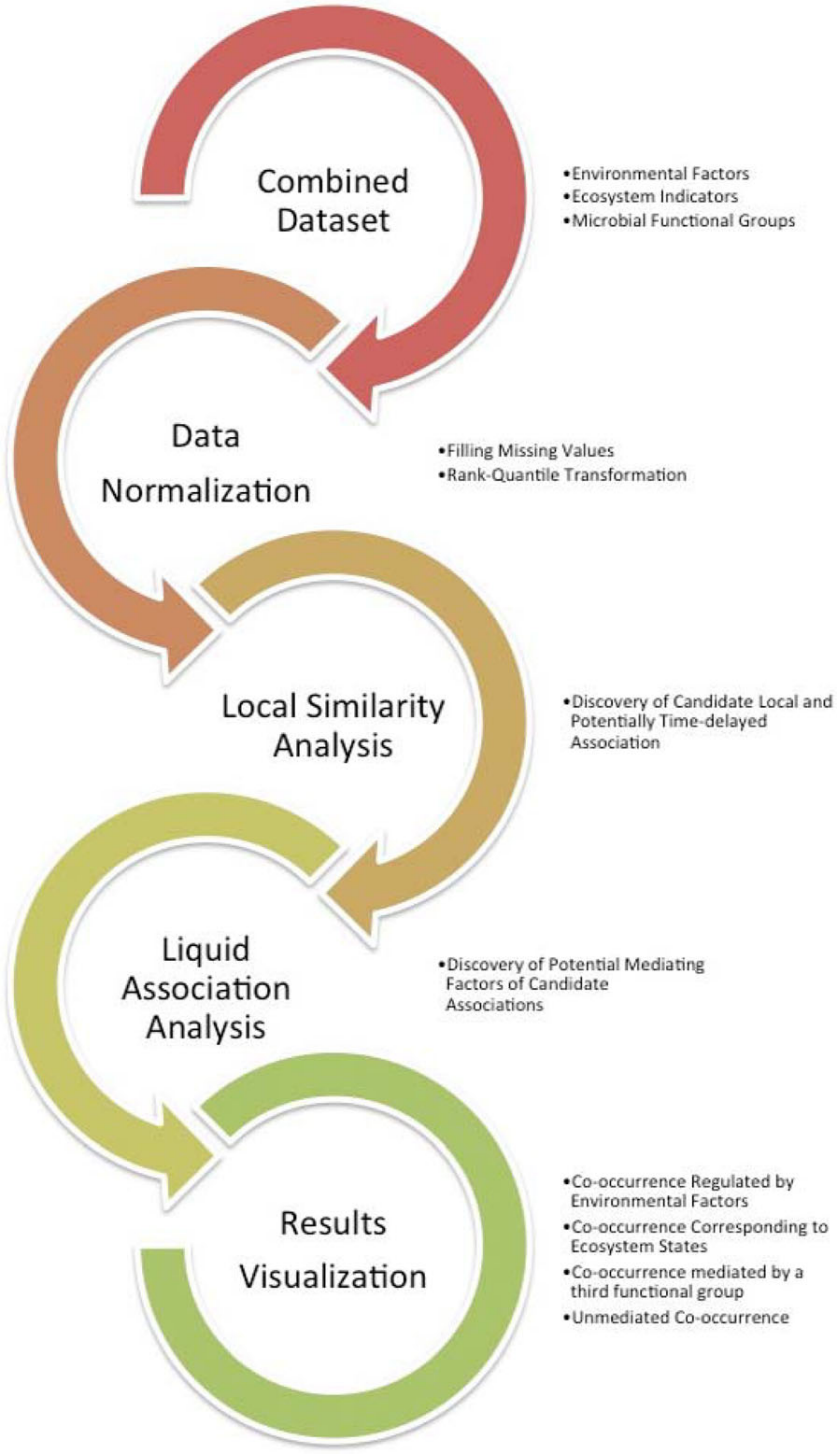
The flowchart of integrated co-occurrence (Local Similarity) and mediation (Liquid Association) analysis pipeline

Our entire process, from data collection through visualization was outlined in Fig. 1 and is detailed in the following subsections. Briefly, we first collected the combined *SPOT* dataset (as detailed in ***Data Collection***) including both operational taxonomic unit (OTU) abundance levels and environmental factors co-measured. We then imputed the missing values and applied rank-based normalization to the dataset (as detailed in ***Data Normalization***). The normalized data was analyzed by the ELSA software to discover significant local pairwise associations (as detailed in ***Local Similarity Analysis***); these are associations that are present over part, though not necessarily all of the time series. Finally, we analyzed these significant local associations with the Extended Liquid Association routines within ELSA to reveal third party meditations of these pairwise interactions (as detailed in ***Liquid Association Analysis***). We finally described a novel visualization scheme to represent mediated association triplets in *Cytoscape* [32], and discussed their implications (as detailed in ***Visualization***).

Our new bioinformatics tool - *Extended Liquid Association (*ELA*)* implements every step described in this analysis. This tool integrates two established and validated methods: Liquid Association analysis by *Li* et al [26] and the Local Similarity Analysis by *Ruan* et al. [6] and *Xia* et al. [28]. We chose to combine these tools because they behave synergistically with each other -- ELSA can uniquely identify local time-series associations and LA can elucidate those associations that are modulated by a third variable. Furthermore, the two tools, LSA and LA have been individually benchmarked extensively, comparing with other tools by independent studies [33, 34]. Indeed, in a comparison of many tools for correlation based network analysis, *Weiss* et al. [13] recently identified LSA as the best tool for correlation-based ecological time series data analysis.

### Data collection

Our data were from the *San Pedro Ocean Time-series* project, which are publicly available from the *BCO-DMO (Biological & Chemical Oceanography Data Management Office)* websites: <http://www.bco-dmo.org/dataset/537137> (physical and chemical data) and <http://www.bco-dmo.org/dataset/535915> (biological data). The generation of these data has been described previously [35]. The samples were collected approximately monthly from 5-m depth from August 2000 through January 2011. Environmental parameters such as temperature and salinity measurements were measured in situ. Nitrate, nitrite, phosphate and silicate concentrations were measured by auto-analyzer. Bacterial heterotrophic productivity was measured through thymidine and leucine incorporation. Bacterial and viral concentrations were measured by SYBR (Synergy Brands, Inc) green epifluorescence microscopy. We also obtained satellite estimates chlorophyll-A concentration, and surface productivity. Other environmental variables included day length, virus to bacteria ratios, the excess phosphate concentration over Redfield ratios, and cell doubling time.

Bacterial community composition was determined by the *ARISA* fingerprinting method in conjunction with clone libraries. The *ARISA* data we used were a set of 423 of operational taxonomic units with putative taxonomic identities. The final data set is a 423 by 120 data matrix, where each row is an *ARISA* profile and each column is a time point (each entry value of an *ARISA* profile stands for the percentage of that operational taxon unit (OTU) in the community at the time. A 35 by 120 data matrix of supplementary biotic and abiotic environmental factors and ecosystem status indicators was combined with the *ARISA* data, in which, each row is one environmental factor and each column is the measure of that environmental factor at one time point, to obtain the final merged 458 by 120 raw data matrix.

### Data normalization

We took the approach as in *Li* et al. [26] to normalize the relative abundance for *ARISA* datasets. In detail, to accommodate possible nonlinear associations and the variation of scales within the raw data, we apply the following approach to normalize the raw dataset before our analysis. We use *X*_*i*_ to denote the raw data of the *i*-th time spot of a variable. Assuming we have *n* samples in OTU profile, first, we take

1 $$ {R}_k=\operatorname{rank}\ \mathrm{of}\ {X}_k\ \mathrm{in}\ \left\{{X}_1,{X}_2,\dots, {X}_n\right\} $$

Then, we take:

2 $$ {Z}_k={\Phi}^{-1}\left[\frac{R_k}{n+1}\right], $$

where *Φ* is the cumulative distribution function of the standard normal distribution. We will take *Z* = *Z*_[1 : *n*]_ obtained through the above procedure as the normalization of *X*.

A flowchart for incorporating Liquid Association (LA) with Local Similarity Analysis (LSA) was shown in Fig. 1. First, we used LSA to find candidate local and without time-delayed associations between factors *X* and *Y*. We filtered the results based on *p*-value and q-value. Then, given the significant LSA factors *X* and *Y*, we computed LA score to screen all environmental/OTU factors to discover potential mediating factor *Z*. Next, a permutation test for liquid association was performed and the results were filtered based on p-value and q-value to remove insignificant triplets. Finally, we used the software Cytoscape to visualize the resulted association network.

### Local similarity analysis

In the next step, Local similarity analysis (LSA) was used to screen locally co-occurring pairs for later third-party mediation analysis. The LSA technique has been fully characterized in previous literature and has been successfully applied to the study of the co-occurrence networks within marine ecosystems [6, 7, 28, 29]. In this study, LSA was applied to find local associations with or without time delays. LSA was carried out with the ELSA software package [29] with parameters *‘-d* 0’ and ‘*-p theo*’. The ‘*-d 0*’ flag in this case indicates to the algorithm that we only wanted to identify unlagged associations -- that is, associations of zero time delay. This parameter was chosen because we were only interested in synchronized co-occurrences and thus accepted only local similarity associations without delays. We also specified, with the ‘*-p theo*’ flag that we wanted to estimate the *p*-value with the faster theoretical approximation [28] rather than the slower permutation based approach.

To correct for multiple testing, we use the false discovery rate (*FDR*) or q-value (*Q*). This q-value is defined as the fraction of false positives if a given association is declared as significant [36]. Resulting pairs with “*P*≤0.001”, “*Q*≤0.05” and with an association segment spanning more than 50% of the total sampled time units were selected as correlated pairs. A cut-off for Local Similarity (LS) Score was determined at 0.28 by those p-value and q-value cut-offs, which could be interpreted as an analog of correlation coefficient of the same sample size.

### Liquid association analysis

Next, we applied liquid association (LA) analysis to the each of the correlated pairs of variables to find their mediating factors. Liquid association analysis was originally developed by *K.C. Li et. al.* for characterizing the internal evolution of expression pattern of a pair of genes (*X*,*Y*) depending on a ‘scouting’ (mediator) gene *Z* [26]. In the microbial ecology setting, we analyze ecological functional factors instead of genes. Suppose *X*, *Y*, and *Z* are ecological functional factors and their measurements are standard normal distributed (after a normalization procedure) random variables with mean 0 and variance 1. The liquid association score (LA score) of *X* and *Y* with respect to*Z*, as denoted by LA (*X*; *Y*| *Z*), is defined as LA(*X*; *Y*| *Z*) = E(*XYZ*).

We estimate the LA score using the average product of three properly normalized factors:

3 $$ \mathrm{LA}\left(X;Y|Z\right)=\frac{1}{m}{\sum}_{i=1}^m{X}_i{Y}_i{Z}_i $$

It can be seen that LA(*X*; *Y*| *Z*) = LA(*Y*; *X*| *Z*) = LA (*Z*; *Y*| *X*)*.* Therefore, the LA score is a measurement of dependent association among the three functional factors; however, the score does not tell which two of the three are associated, which other is the mediating the relationship. In our analysis, the LSA approach supplements as the first step to identify the correlated pairs and then we used the identified pairs to scout for mediating factors.

To see whether the LA score computed is statistically significant, a permutation test was performed. To do this, after we computed the true LA score for *X* and *Y* with respect to *Z*, we randomly permuted the sequences of *Z*, and computed again a permuted LA score and compared it to the true LA score to see whether it was more extreme. The procedure was repeated many times (*N* = 1000) and the *p*-value (*P*) was calculated as the fraction that the permuted LA scores were higher than the true LA score. We used “*P*≤0.001” and “*Q*≤0.05” to control for statistical significance and multiple testing.

**Fig. 2:**
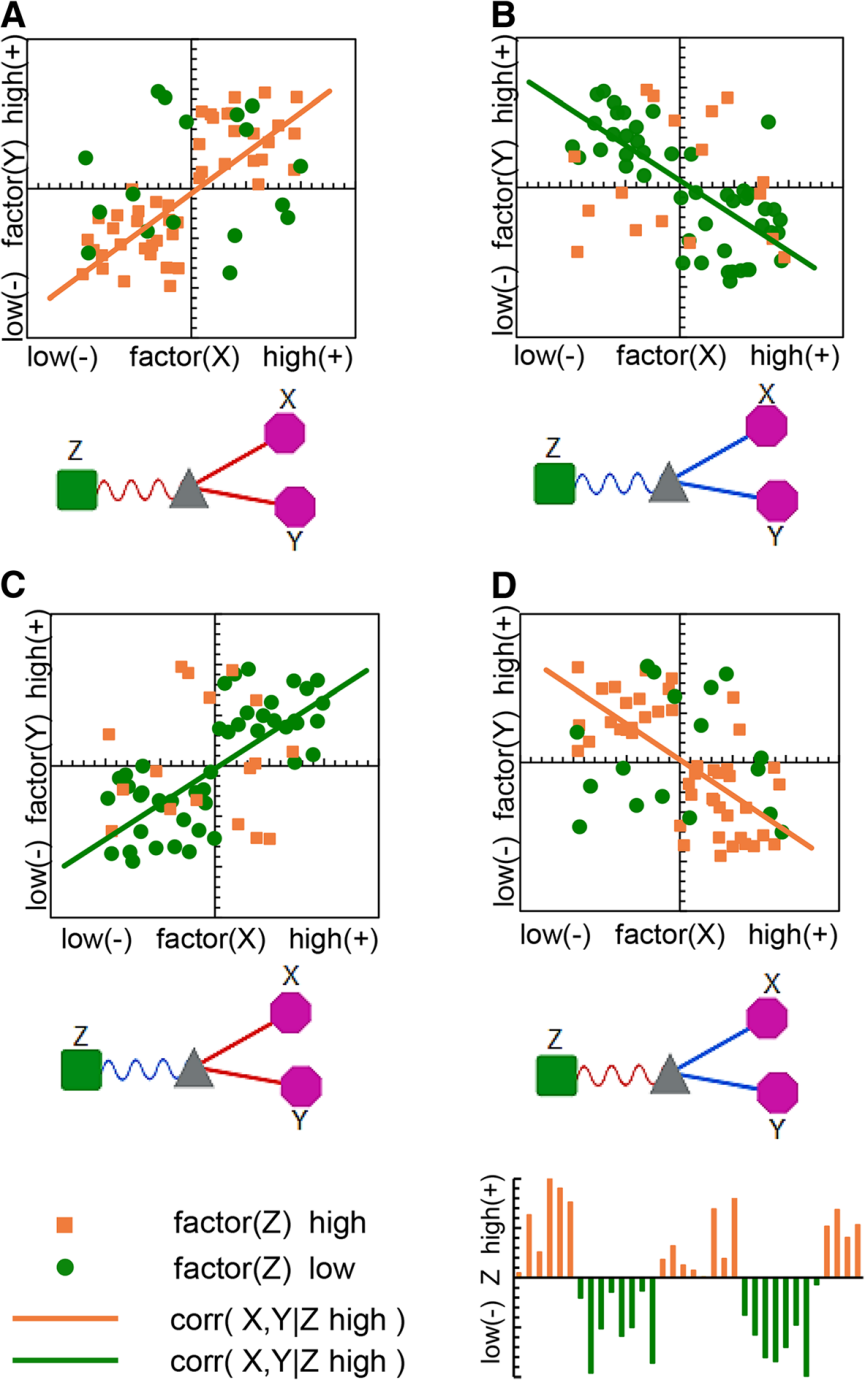
Mediated correlation and example Cytoscape diagrams for all liquid association types of factors *X*, *Y* and *Z*. **a** High *Z* level enhances the positive correlation between *X* and Y; **b** Low *Z* level enhances the negative correlation between *X* and *Y*; **c** Low *Z* level enhances the positive correlation between *X* and *Y*; **d** High *Z* level enhances the negative correlation between *X* and *Y*

As shown in Fig. 2, There are four types of mediated correlation relationship, i.e., liquid association types, among factors *X*, *Y* and Z: (A) High *Z* level enhances the positive correlation between *X* and *Y* (Fig. 2a); (B) Low *Z* level enhances the negative correlation between *X* and *Y* (Fig. 2b); (C) Low *Z* level enhances the positive correlation between *X* and *Y* (Fig. 2c); (D) High *Z* level enhances the negative correlation between *X* and *Y* (Fig. 2d). In all cases, the correlation between *X* and *Y* is mediated by the level of *Z*. Those liquid association scenarios include cases when factors *X* and *Y* are correlated in one direction when *Z* is in one state, and *X* and *Y* cease to correlate or even to correlate reversely when the state of *Z* changes [26].

For example, the scenario a high *Z* level enhances the positive correlation between *X* and *Y* is shown in Fig. 2a. In the upper panel of Fig. 2a, factors *X* and *Y* were displayed in a scatter plot and the corresponding status of *Z* was both shape- and color-coded in the scattered points. A green circle stands for a low level of *Z* while an orange square stands for elevated level. The mediated co-occurrence is represented by an orange-colored regression line applied to the scatter points of high level of *Z* only. We designed the graphic element as in the lower panel of Fig. 2a to illustrate this relationship for visualizing the co-occurrence networks using Cytoscape. Those three factors: *X*, *Y* and *Z,* were interconnected via a gray triangle to denote this three-way association. The wavy line connecting the triangular with the factor *Z* stands for mediation and the solid lines connecting the triangular with *X* and *Y* stands for the mediated correlation. We use red to indicate positive correlations and blue for negative correlations. Similarly, one can interpret the other types of liquid associations as depicted in the other panels of Fig. 2.

### Visualization

To illustrate those complex triplet relationships in Cytoscape, we created a representation diagrams for each type: Green squares represent environmental factors or ecosystem status indicators. Violet octagons represent functional groups (OTUs). Gray triangles represent the three-way liquid association. Solid lines indicate local similarity associations - red solid lines indicate positive correlations while blue solid lines indicate the negative. Wavy lines indicate third-party mediation - red wavy lines indicate that the association is strong when the mediating variable is high, while the blue wavy lines indicate that the association is strong when the mediating variable is low (see Fig. 2).

We loaded all liquid association and local similarity connections into Cytoscape [32], along with metadata of bacterial and environmental factors. With Cytoscape we illustrated all significant three-way associations that involved environmental parameters. Those associations were all statistically significant and their pairwise *LS* scores were higher than 0.28. We went on to investigate associations that involved nodes of special interest. We first investigated pairwise interactions between operational taxonomic units that were regulated by environmental parameters. We also investigated associations that involved at least one OTU from the *SAR11* cluster, as well as associations that contained at least one OTU from the class *Flavobacteria*.

## Results

### Co-occurrence mediated by environmental factors

#### Bacterial abundance

**Fig. 3:**
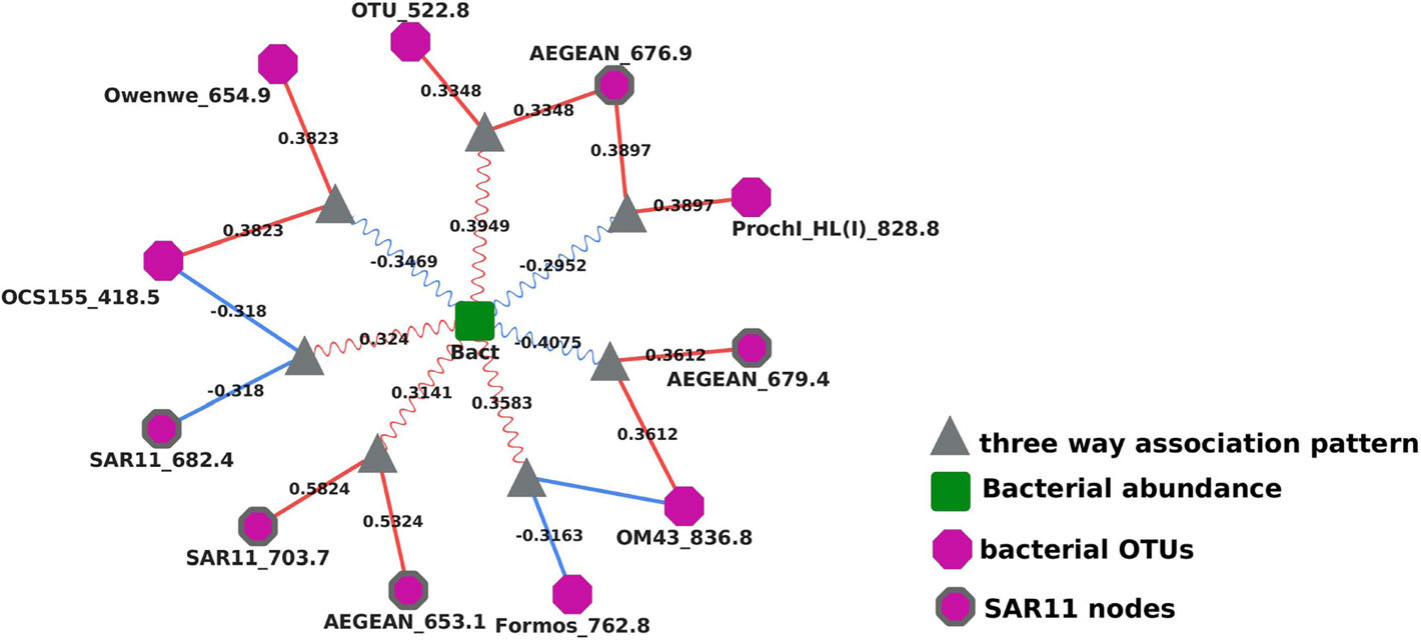
A sub-network of local and liquid associations in which OTU correlations were mediated by the total bacterial abundance (*Bact*), an environmental factor

Inspection of the liquid association interactions between pairs of OTUs that were mediated individual environmental parameters identified a variety of three-way interactions. Bacterial abundance (*Bact*) appeared to predict correlations of seven pairs of operational taxonomic units (Fig. 3). That is, seven pairs of positively and negatively correlated OTUs showed strongest correlations in their relative abundance in some cases when total bacterial abundance was high, and in other cases when bacterial abundance was low. For instance, the correlation between *AEGEAN_676.9* and *OTU522.8* is positive and was at its strongest when the total bacterial abundance was high. Conversely, as shown in the figure, the correlation between *AEGEAN_676.9* and *Prochl_HL (I)_828.8* was positive and at its strongest when total bacterial abundance was low.

In total, five of those seven pairs of bacteria (*SAR11_682.4, SAR11_703.7, AEGEAN_653.1, AEGEAN_679.4, AEGEAN_676.9*) contained at least one OTU from the *SAR11* cluster. Furthermore, six of the seven pairs were linked by a common OTU (*Bact*). That is, there were two OTUs (*OCS155_418.5* and *OM43_836.8*) that were correlated (positively or negatively) with another OTU when total bacterial abundance was high but correlated with a different OTU when the bacterial abundance was low. Overall it suggested that an alternating pattern exists for those common OTUs as they change interacting partners and types as the total bacteria abundance rise or drops, for example, when *Bact* is high *OM43* may compete with *Formos* but when *Bact* is low, *OM43* instead cooperates with *AEGEAN*.

### Other environmental factors

**Fig. 4:**
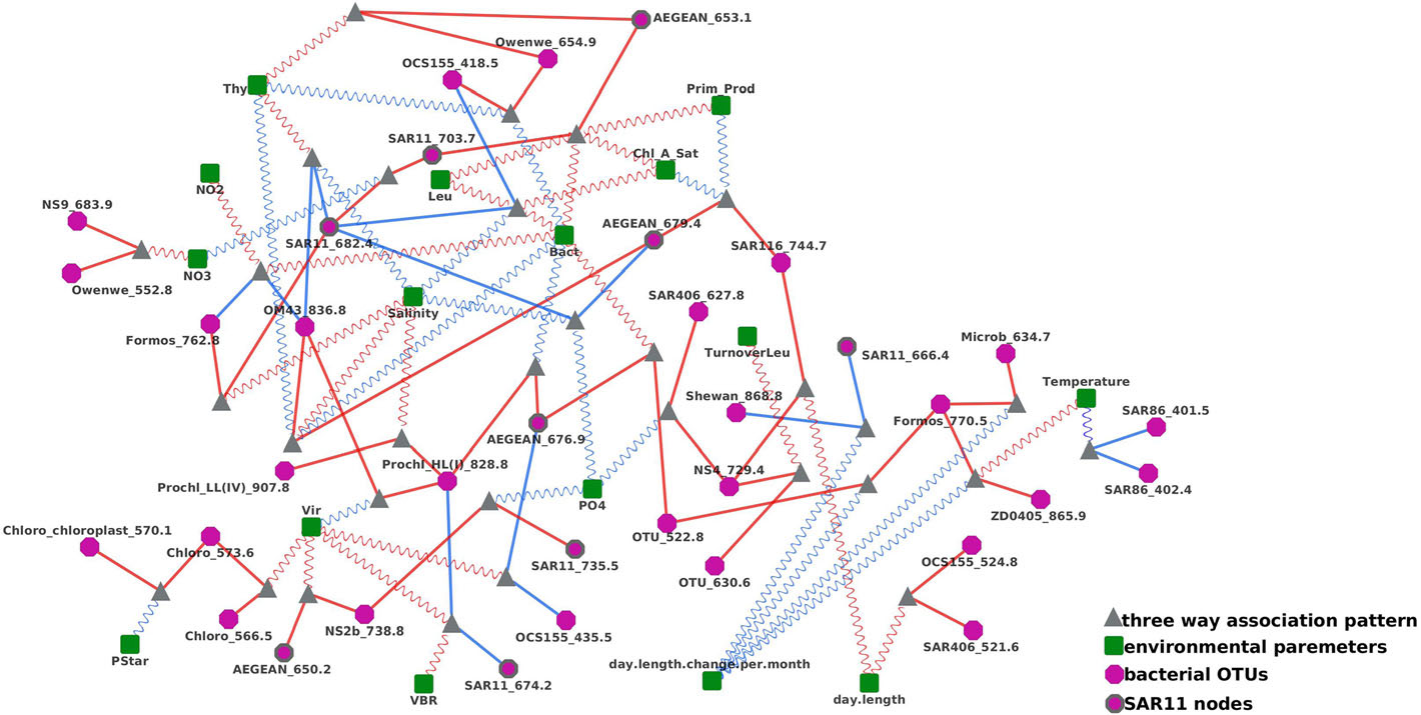
A comprehensive network of local and liquid associations in which OTU correlations were mediated by environmental factors

There were many other environmental parameters that also appeared to be in liquid association with correlated OTU pairs, including silicate (12 connections), viral abundance (5 connections), salinity (6 connections), bacterial productivity as measured by thymidine incorporation (4 connections), the rate of change in day length (spring vs fall) (4 connections), phosphate concentration, and chlorophyll-A concentration (3 connections) (see Fig. 4).

For example, chlorophyll-A concentration (*Chol_A_Sat*) was a critical mediator of alphaproteobacteria and actinobacteria clades. When it was high, it enhanced the cooperation between *SAR11_703.7* and *AEGEAN169_653.1* clades - both were alphaproteobacteria, to promote carbon oxidization. This positive triplet association indicated likely oxidative bacteria bloom after a major increase of marine productivity from photosynthetic plankton as indicated by high *Chol_A_Sat* level. Such alphaproteobacterial cooperativity however was inhibited by competition from OTU *OCS155_418.5*, which was a predominantly actinobacteria clade and was also involved in degrading organic matters, as we could observe an enhanced negative correlation between *SAR11_682.4* and *OCS155_418.5* when *Chol_A_Sat* concentration was high. A nature explanation for the observation was alphaproteobacteria and actinobacteria’s competing biochemical nature to degrade organic matters. Interestingly, when *Chol_A_Sat* was low, it also enhanced the cooperation between two other alphaproteobacterial OTUs (*SAR216_744.7* and *AEGEAN_679.4*). This partner switch suggested subclones of alphaproteobacterium clades could rise or cease to dominate clade activity depending on changing environmental status.

### Co-occurrence mediated by other operational taxonomic units

#### SAR11

**Fig. 5:**
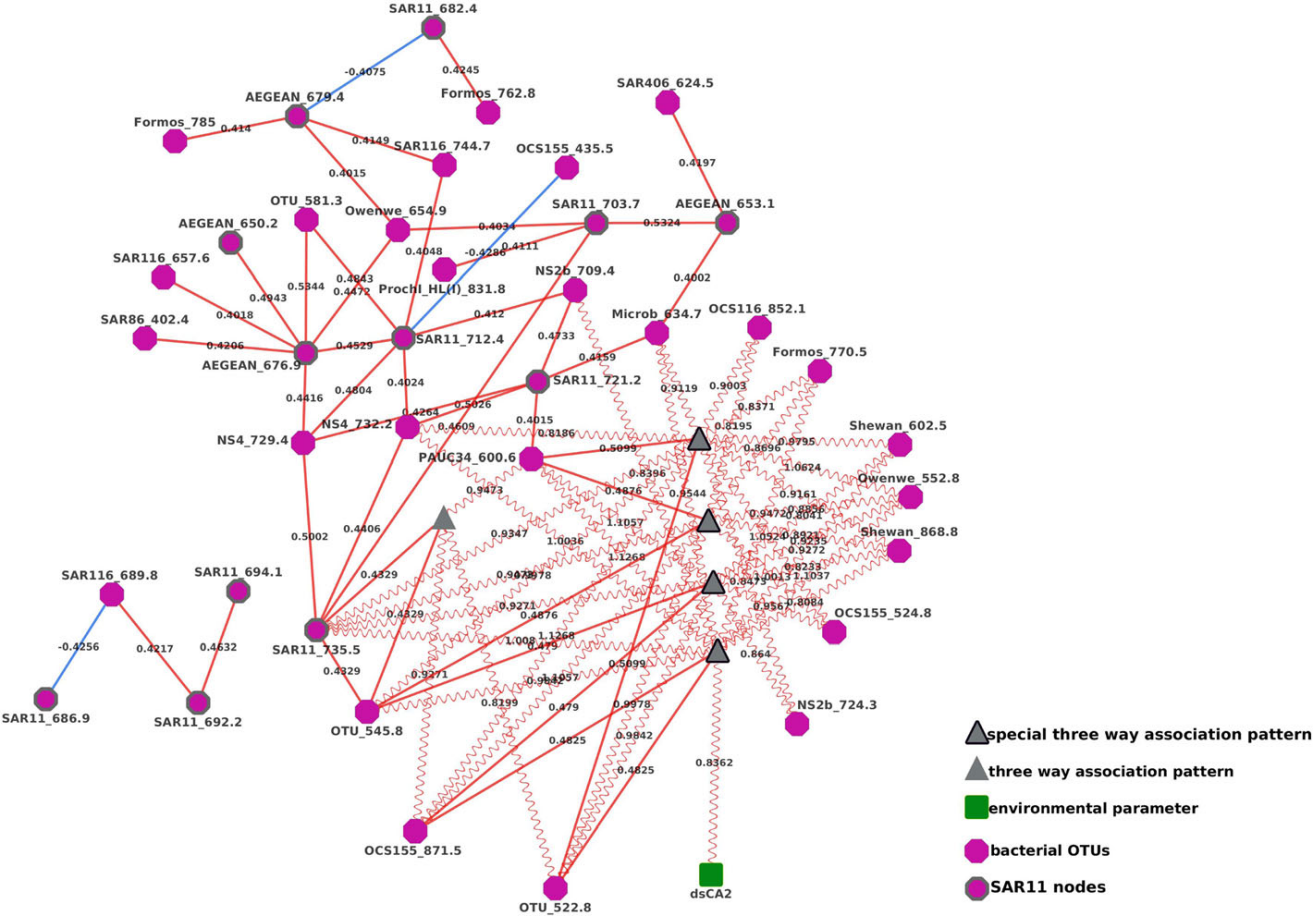
A sub-network showing OTUs mediated and/or correlated to/by *SAR11* OTUs. SAR11 nodes were highlighted by bold outlines. All *SAR11* in the dataset and their significant liquid and local associations were shown

Due to its unique metabolism and ecological role [37, 38], as well as its abundance and temporal variability in the surface of the san pedro channel [6] and globally [39], we focused on three-way associations in which any of the three nodes were from the *SAR11* clade (*Pelagibacterales*). It was evident that while many *SAR11*s were correlated with other operational taxonomic units, only OTU *SAR11_735.5* appeared to be involved in (several) three-way interactions with cutoffs of a liquid association effect larger than 0.8 (Fig. 5).

**Fig. 6:**
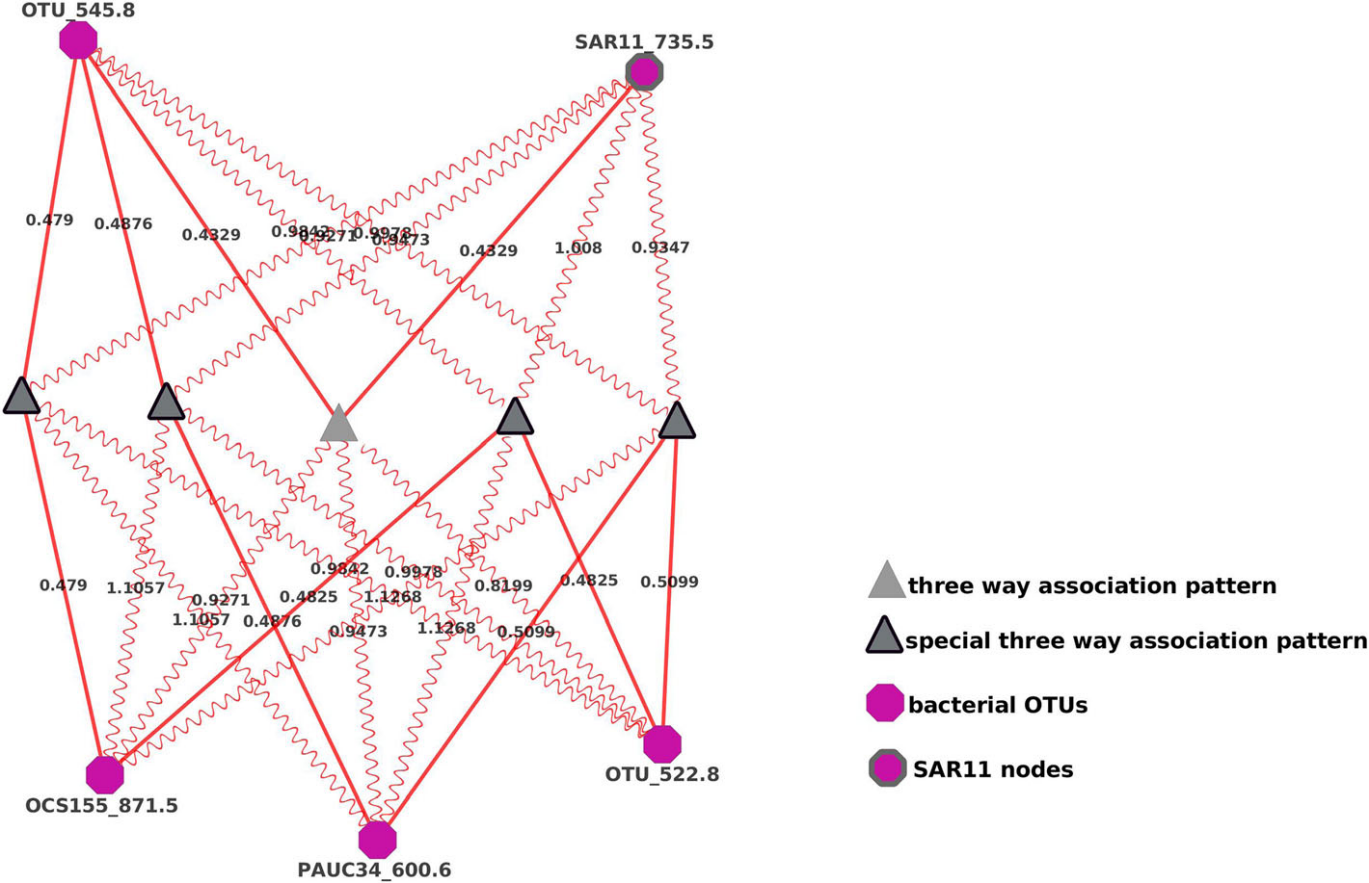
A sub-network of four bacterial OTUs that were liquid-associated with *SAR11* and *SAR11_735.5*

In the sub-network formed by the five correlating OTUs (Fig. 6), *SAR11_735.5* has a special status. First, *SAR11_735.5* and *OTU_545.8* (an OTU of unknown taxonomy) were positively correlated. They were connected by red solid lines and their correlations were mediated (gray triangle) by three OTUs (*OCS155_871.5*, *PAUG3G_600.6*, and *OTU_522.8*). When the abundance of those three mediating OTUs were high, the strength of the positive correlation between *SAR11_735.5* and *OTU_545.8* was stronger, and those positive three-way interactions were represented by red wavy lines. Further, the correlations of four pairs of *SAR11* OTUs (*OTU_545.8* and *OCS155_871.5*, *OTU_545.8* and *PAUC34_600.6*, *OCS155_871.5* and *OTU_522.8*, *PAUC34_600.6* and *OTU_522.8*) were mediated by *SAR11_735.5*. When the abundance of *SAR11_735.5* was high, the strength of these four correlations were stronger. Notably, all correlations between pairs of OTUs and three-way interactions in this network were all positive, implying these OTUs benefited from each other’s boom and their relationships belonged to mutualism. This subgraph illustrated a complex and largely feed-forward trophic network that whose dynamics depended on the activity of *SAR11* clades.

#### Flavobacteria

**Fig. 7:**
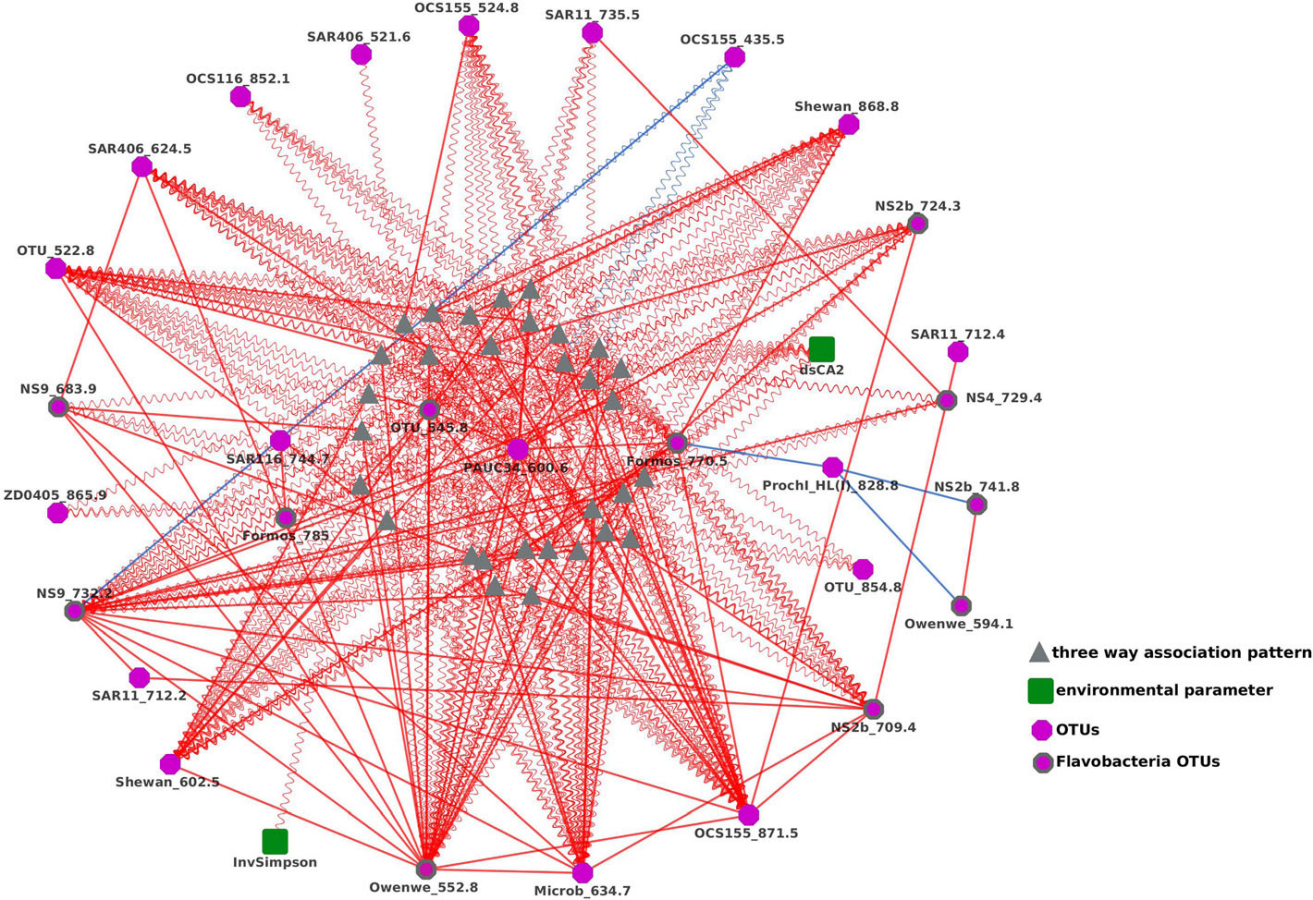
A sub-network showing OTUs mediated and/or correlated to/by *Flavobacteria*. *Flavobacteria* nodes were highlighted by bold outlines

The genus that appeared to be involved in the most three-way interactions was Flavobacteria with four nodes appearing in a tight cluster with other OTUs, most of which were liquid-associated (Fig. 7). These associations suggested that the bacteria in the cluster occasionally co-occurred together. At other times the Flavobacteria and their cooperatives were not co-occurring, but they were not necessarily all absent.

There were 31 liquid association patterns in the (Fig. 7). All the Flavobacteria OTUs, which were highlighted by bold outlines, were involved in triple associations. *OTU_522.8* was a special OTU (which was located at the left of Fig. 7) in this cluster. It was involved in more than 30 three-way association patterns. For instance, *OTU_522.8* was positively correlated with *OTU_545.8*, and their correlation was mediated by six OTUs, which included *Formos_785* and *NS9_683.9*. The red solid and wavy lines indicated that the relationship of those OTUs was mutualism. The correlation between *OTU_522.8* and *OCS155_871.5* was mediated by *OCS155_435.5*, the blue wavy line indicated that when the abundance of *OCS155_435.5* was high, the positive correlation between *OTU_522.8* and *OCS155_871.5* was weaker, which demonstrates a competitive relationship between *OCS155_435.5*, *OTU_522.8* and *OCS155_871.5*.

## Discussion

The interactions between microbial species are complex and often non-linear. Some of those interactions are regulated by factors within the microbial community itself [40], such as the levels of key organic matter producers. Some of them are however mediated by environmental factors, such as temperature, oxygen, among others. For example, water column mixing can disturb microbial communities by disrupting the physical–chemical gradients created by thermal stratification known to define niches for microorganisms [41–45]. Liquid association is thus promising in revealing and elucidating these complex non-linear co-mediated associations.

### Environment

Analysis of the SPOT dataset suggested that there were a series of correlations that occurred most strongly only under certain conditions. We observed that several biotic and environmental parameters, especially bacterial and viral abundance, silicate concentrations and salinity appeared to predict a number of correlations between bacteria. Liquid associations involving these parameters suggest that not only do bacteria interact with each other and environmental parameters, but furthermore that the nature of the relationships between many bacteria are likely affected by their environment.

Liquid associations in which bacterial abundance mediated the relationships between pairs of OTUs could indicate symbioses or competitive interactions between species that depend upon either the overall density of the bacterial community. Denser microbial communities, for instance are likely to have higher encounter rates between bacteria pair of bacteria which could potentially increase opportunities for symbioses, antagonistic interactions, viral exchange and other interactions. Alternatively, some unmeasured parameter, such as predation [46]. For instance, the *AEGEAN_676.9* bacteria (which is a SAR11 relative [47], is positively associated with *OTU_522.8* under high bacterial abundance, and with *Prochlorococcus High Light Strain 828.8* under low bacterial abundance. SAR11 is known to require metabolic by products of other organisms, and one could expect that it gathers these metabolites from different organisms under different conditions.

Viruses are believed to have complex effects on bacterial communities, and this study suggests a number of interactions that may be mediated either by the viruses themselves or an unmeasured factor that relates to viral abundance [48, 49]. Silicate concentrations likely affect the protest community with diatoms utilizing silicate and dinoflagellates taking advantage of low silicate conditions; these eukaryotes likely interact with bacteria by producing chemicals, consuming resources, through predation and possibly through symbioses [50]. These different conditions might impact the kinds of relationships bacteria could have. For instance, the several bacteria that work together to break down a compound produced by one source might show up as a three-way positive interaction. Meanwhile two bacteria that compete to colonize a diatom symbiotic host or diatom carcass might show up as a three-way negative interaction with diatoms.

The parameter in our dataset that appeared to predict the associations between the most of OTUs was the number of days that had elapsed since the beginning of our data set. This suggests that a number of correlations that could be found in the first part of our data set were not seen in the later years of the data set and vice versa. This suggests a long-term temporal shift in the kinds of associations in the bacterial community.

### OTUs

There were numerous cases of interactions between trios of bacteria and noticed that certain groups appeared to be associated with these three way interactions but not others. We observed notably different patterns between the liquid associations that contained at least one *SAR11* OTU and those liquid associations that contained at least one *Flavobacterial* OTU. *SAR11*s, with the exception of one OTU (*SAR11_735.5*), appear to not be found in three-way liquid associations in this dataset, meaning that while they may correlate with other bacteria, the magnitude of this correlation is rarely associated with the relative abundance of a third OTU. This lack of three way associations suggests that *SAR11* bacteria themselves do not influence associations between other kinds of bacteria. On the other hand, out of ten *Flavobacterial* OTUs that correlated with other bacteria, eight of these appeared to be part of liquid associations. The overall pattern for *Flavobacteria* is that they were part of a cluster of positive liquid and local associations. This suggests that there was a set of conditions in which *Flavobacteria* and related OTUs co-occurred positively. However, under other conditions, these OTUs were un-related to each other. Likely these *Flavobacteria* and their associates were all responding to some unmeasured environmental situation such as a bloom. That is, when the unmeasured condition was happening, one set of associations predominated, and when it was not happening another set predominated. This manifests as three way associations between OTUs. Another possibility is that communities may structure themselves in different ways, with different sorts of exchanges between organisms. Under one structure, we see one set of patterns and under another we see a different set of patterns. This structure could be driven by (unmeasured) environmental variability, could be a process shaped the interplay between organisms themselves. As no environmental parameters that we measure appeared to associate with this cohort, we can only speculate at this time what likely caused this association.

This difference in patterns between microorganisms suggests different trends in the ecological associations between organisms of different genetic makeup. *Flavobacteria* are known for being particle associated and able to break down complex molecules and might respond to situations in which particles and unusual sources of dissolved organic material are prevalent, such as after a plankton bloom [51]. *SAR11*s on the other hand are known as oligotrophic chemoheterotrophs with inflexible metabolisms. Accordingly, their relationships with other organisms may be less governed by environmental or biological factors.

### Caveats

It should be noted that the collected data and obtained results are only associations (not causality), and need be further verified their validity by one by one OTU experimentation. Furthermore, the validity of ELA depends on the goodness of community fingerprint data, such as number of samples, accurate abundance of each species et.al. In the future, by applying ELA. In the future, by applying liquid association analysis to an integrated community big dataset, it is possible to find resilient associations are common to all communities as well as associations unique to certain community types. This can help us better understand the ecological dynamics with regard to community differences.

## Conclusions

Multi-party correlations have been vastly revealed between biological entities ranging from ecosystems to genes [52]. Our approach of coupling local association with liquid association allows additional insight by first identifying statistical patterns, and then using liquid association to identify whether the strength of those patterns is modulated by additional variables. This approach examines many associations at once, and is thus inherently hypothesis generating. It allows for an initial exploration of patterns in a microbial data-set that go beyond pairwise correlations to three way correlations. In our analysis of the San Pedro Ocean time series, this approach has shown us for the first time that associations between organisms appear to change from the beginning to the end of the data set, that bacterial and viral total abundance appears to modulate the interactions between relative abundances of individual OTUs, and that OTU associations appear to be modulated both by environmental parameters and by other OTUs. These patterns provide a starting point for future observational approaches to explore whether these sorts of three-way patterns are robust across ecosystems, and for experimental and modeling approaches to explore the mechanistic underpinnings of these sorts of associations. We think this approach will be valuable in the analysis of any multivariate time-series data sets. We encourage groups to use the freely available software which has been added as an extension to the ELSA software package [28, 29] whose source code can be found at http://bitbucket.org/charade/elsa.

## Abbreviations

ARISA: Automated ribosomal intergenic spacer analysis
ELSA: Extended local similarity analysis
FDR: False discovery rate
LA: Liquid association
LSA: Local similarity analysis
OTU: Operational taxonomic unit
SPOT: San Pedro Ocean Time Series
